# Late diagnosis of Gitelman syndrome in an elderly patient: identification of a novel *SLC12A3* pathogenic variant

**DOI:** 10.1590/2175-8239-JBN-2026-0105en

**Published:** 2026-09-14

**Authors:** Lígia Ribeiro, Micael Pompermayer, Catarina Santos, Joana Coutinho

**Affiliations:** 1 Unidade Local de Saúde de Castelo Branco, Serviço de Nefrologia, Castelo Branco, Portugal.

**Keywords:** Gitelman Syndrome, Hereditary tubulopathy, Hypokalemia, Genetic Testing.

## Abstract

Gitelman syndrome (GS) is an inherited salt-wasting, hypokalemic tubulopathy caused mainly by biallelic loss-of-function variants in the *SLC12A3* gene. Despite being classically diagnosed in adolescence, delayed recognition may occur. We present the case of a 74-year-old man with a history of hypokalemia, in whom the diagnostic evaluation revealed hypokalemia, hypomagnesemia, metabolic alkalosis, hypocalciuria, and secondary hyperreninemic hyperaldosteronism, consistent with renal potassium wasting. Given the late presentation, the main challenge was the need to distinguish an inherited tubulopathy from common acquired causes of hypokalemia. Genetic testing was performed, identifying a previously unreported homozygous out-of-frame deletion in exon 25 of the *SLC12A3* gene, predicted to result in loss of protein function and therefore to be pathogenic. Treatment with supplementation plus spironolactone achieved sustained biochemical improvement and clinical stability during long-term follow-up. This case highlights that GS should be considered in patients with chronic unexplained hypokalemia regardless of age, and that genetic testing is essential for definitive diagnosis.

## Introduction

Gitelman syndrome (GS) is an autosomal recessive salt-wasting tubulopathy charac­terized by hypokalemia, metabolic alkalosis, hypomagnesemia, hypocalciuria, and secondary hyperreninemic hyper­aldosteronism^1,2^. It is considered the most common inherited tubulopathy, with an estimated prevalence ranging from 1 in 10,000 to 1 in 40,000 individuals, although the true prevalence may be underestimated because mildly affected patients frequently remain undiagnosed^1^. The disorder is most often caused by biallelic pathogenic variants in the *SLC12A3* gene, which encodes the thiazide-sensitive sodium-chloride cotransporter (NCC) located on the apical membrane of distal convoluted tubule cells^1,3^.

Loss of NCC activity impairs sodium and chloride reabsorption, leading to mild extracellular volume contraction. This chronic salt wasting activates the renin-angiotensin-aldosterone system (RAAS), promoting distal sodium reabsorption in exchange for potassium and hydrogen ion secretion and thereby resulting in hypokalemia and metabolic alkalosis^2,4,5^. Associated hypomagnesemia is thought to be secondary to altered magnesium transport, particularly through downregulation or dysfunction of TRPM6-mediated magnesium reabsorption in the distal convoluted tubule^3,6^. Hypocalciuria, another hallmark of GS, likely reflects increased proximal tubular calcium reabsorption in response to mild volume depletion and altered distal tubular transport^3^.

The phenotype of GS is heterogeneous. Some patients are essentially asymptomatic and are diagnosed only after incidental laboratory abnormalities are detected, whereas others develop fatigue, muscle weakness, cramps, salt craving, thirst, nocturia, paresthesias, tetany, rhabdomyolysis, chondrocalcinosis, or cardiac arrhythmias^7,8^. The severity of manifestations depends not only on the degree of electrolyte disturbance but also on age, dietary intake, comorbid conditions, intercurrent illness, and the specific genetic background of the patient^1,7^. Although GS is often diagnosed in adolescence or early adulthood, delayed diagnosis may occur, particularly in individuals with mild symptoms or fluctuating biochemical abnormalities.

In clinical practice, persistent hypokalemia is far more often attributed to acquired conditions than to inherited tubulopathies. Gastrointestinal losses, diuretic or laxative use, endocrine disorders, and secondary renal causes are usually considered first^8^. As a result, GS may not be suspected until a characteristic biochemical constellation becomes evident. This is especially true in older adults, in whom clinicians may be less likely to consider an inherited disorder. The differential diagnosis also includes Bartter syndrome and other renal tubular disorders, requiring careful interpretation^9^.

This report uses the case of an elderly patient with long-standing unexplained hypokalemia to illustrate a structured clinicopathological discussion focused on diagnostic reasoning, relevant differential diagnoses, clinicopathological correlation, and therapeutic implications. It also expands the mutational spectrum of GS by describing a previously undescribed *SLC12A3* variant.

## Diagnostic Reasoning

A 74-year-old obese man with a BMI of 31.2 kg/m^2^ and a medical history of dyslipidemia and atrophic gastritis was referred for an internal medicine consultation after moderate hypokalemia (serum potassium, 2.6 mmol/L) was detected during an emergency department visit for acute vomiting attributed to presumed viral gastroenteritis. His regular medication included spironolactone, tamsulosin, and rosuvastatin. A review of previous records showed that mild hypokalemia had been documented repeatedly in primary care over the previous 10 years. At the time of the outpatient evaluation, the vomiting had resolved completely. Although acute gastrointestinal losses could account for transient potassium depletion, they could not explain a decade-long pattern of persistent hypokalemia. The patient described long-standing thirst and nocturia, but denied muscle weakness, cramps, paresthesias, palpitations, syncope, or episodes suggestive of tetany. There was no history suggestive of eating disorders, diarrhea, laxative abuse, or surreptitious diuretic use. The exclusion of surreptitious use of laxatives and diuretics was based on the clinical history and the absence of any prescription for loop or thiazide diuretics in the patient’s medication records over the preceding decade, combined with the persistently low urinary chloride excretion (92 mmol/24 h, below the lower limit of normal [110 mmol/24 h]), which would be atypical for active diuretic use. Family history was unremarkable.

The first diagnostic step was to distinguish between extrarenal and renal potassium loss. In a patient with chronic hypokalemia, metabolic alkalosis immediately raises several possibilities, including vomiting, diuretic use, mineralocorticoid excess, and inherited salt-wasting tubulopathies^8^. The lack of ongoing gastrointestinal symptoms, the chronicity of the abnormality, and the absence of clear drug-related potassium loss made renal potassium wasting more likely.

Laboratory evaluation under usual medication confirmed persistent hypokalemia and hypo­magnesemia, together with metabolic alkalosis. The results are presented in Table 1. Renal function and thyroid function were normal. Plasma renin and aldosterone levels were elevated, indicating secondary hyperreninemic hyperaldosteronism rather than primary aldosterone excess. This profile strongly suggested chronic sodium wasting with effective volume depletion.

**Table 1:** Laboratory findings at admission of a patient with GS

Laboratory test (units)	Obtained values	Reference
Arterial Blood Gas		
pH	7.421	7.350–7.450
pO_2_ (mmHg)	88	75–100
pCO_2_ (mmHg)	36.8	35–45
Potassium (mmol/L)	3.31	3.5–4.5
Calcium (mmol/L)	1.27	1.15–1.30
Bicarbonate (mmol/L)	23	18–22
Biochemistry and Hematology		
Hemoglobin (Hb)	16.7	13.0–17.0
Leucocyte count (10^3^/uL)	8.29	4.0–10.0
Platelets (10^3^/uL)	298	150–400
Serum Urea (mg/dL)	33.3	19.3–49.3
Serum Creatinine (mg/dL)	0.77	0.70–1.30
Sodium (mmol/L)	137	136–145
Potassium (mmol/L)	3.4	3.6–5.1
Magnesium (mmol/L)	1.49	1.60–2.60
Thyroid-Stimulating Hormone (µUI/mL)	1.68	0.40–4.40
Cortisol (ng/dL)	22	4.30–22.40
Adrenocorticotropin Hormone (pg/mL)	24	09–52
Active Renin (µU/mL)	278	07–76
Aldosterone (pg/mL)	1355	40–310
24-hour urine collection		
Volume (mL)	2400	–
Potassium (mmol/24 h)	127.2	25.0–125.0
Sodium (mmol/24 h)	88.3	40.0–220.0
Chloride (mmol/24 h)	92	110.0–250.0
Calcium (g/24 h)	0.06	0.10–0.30

A 24-hour urine collection showed hypocalciuria, with a fractional excretion of calcium of 0.0045%. This finding was particularly informative. In the setting of hypokalemic metabolic alkalosis, hypocalciuria is classically associated with GS and helps distinguish it from Bartter syndrome, in which urinary calcium excretion is usually normal or increased^9^. The presence of hypomagnesemia further supported the diagnosis of GS, since magnesium depletion is frequent and often prominent in this disorder^2,3^.

At this stage, the most probable diagnosis was Gitelman syndrome. However, alternative explanations still required exclusion. Primary hyperaldosteronism was unlikely because renin was elevated rather than suppressed. Cortisol levels did not support the diagnosis of hypercortisolism. Renal artery stenosis, which can also lead to hyperreninemic hyperaldosteronism, was excluded by abdominal computed tomography with angiography. No adrenal or other intra-abdominal mass was identified. Imaging also showed no nephrocalcinosis, a finding that, if present, might have pointed toward alternative tubular disorders or long-standing mineral imbalance.

Electrocardiography showed sinus rhythm, and transthoracic echocardiography demonstrated only mild aortic insufficiency. These findings were reassuring in view of the risk of arrhythmia associated with chronic hypokalemia, but they did not diminish the need to clarify the underlying cause.

Taken together, the persistent hypokalemia, metabolic alkalosis, hypomagnesemia, hypocalciuria, normal blood pressure, and secondary hyperreninemic hyperaldosteronism formed a highly coherent biochemical pattern consistent with GS. Because the presentation was unusually late and no family history was available, genetic confirmation was pursued both to secure the diagnosis and to better characterize the underlying defect.

After informed consent was obtained, next-generation sequencing using an Illumina platform identified a previously undescribed homozygous out-of-frame deletion of exon 25 in the *SLC12A3* gene: c.(2883+1_2884-1)_(2951+1_2952-1)del. The variant was absent from population databases, including gnomAD and the Database of Genomic Variants. Given its predicted disruptive effect on the reading frame and consequent loss of normal protein function, it was considered pathogenic and confirmed the diagnosis of Gitelman syndrome. Family screening was not performed because the patient had no first-degree relatives available for evaluation.

This biochemical profile remained stable over a 5-year follow-up period, as presented in Table 2, with no neuromuscular or cardiovascular complications observed and stable blood pressure measurements.

**Table 2:** Annual values of serum potassium, magnesium, and creatinine over a 5-year follow-up

	Serum potassium (mmol/L)	Serum magnesium (mg/dL)	Serum creatinine (mg/dL)
Year 1	3.60	1.22	0.79
Year 2	3.68	1.30	0.81
Year 3	3.63	1.31	0.80
Year 4	3.71	1.24	0.79
Year 5	3.68	1.28	0.80

## Clinical Challenges

This case illustrates several reasons why GS may remain unrecognized for many years. Although GS is a monogenic disorder, its clinical expression ranges from almost silent biochemical abnormalities to symptomatic disease with substantial impairment, reflecting a high phenotypic variability^1,7^. In this patient, the absence of overt neuromuscular or cardiovascular complaints likely contributed to a low degree of clinical suspicion for many years.

Because GS is traditionally associated with younger patients, clinicians may not readily consider it in an older adult presenting with chronic hypokalemia. Yet the genetic defect is present lifelong, and the age at diagnosis depends more on symptom burden and diagnostic awareness than on disease onset.

Other challenges involved the interpretation of urinary and biochemical data in the setting of treatment. The patient was already receiving spironolactone at the time of evaluation, and subsequent supplementation also influenced follow-up measurements. In real-world practice, investigations are often performed under nonideal conditions, and diagnostic reasoning must integrate imperfect data rather than rely on a single unconfounded test result. Spironolactone may have partially corrected potassium levels, blunted the aldosterone response, and further stimulated renin secretion through reactive hyperreninemia. Despite this, the overall biochemical pattern, characterized by persistent hypokalemia, hypomagnesemia, hypocalciuria, and elevated renin with secondary hyperaldosteronism, remained highly consistent with Gitelman syndrome. Importantly, the diagnosis was ultimately secured by molecular confirmation, which is independent of pharmacological confounders. This limitation is particularly relevant, and it should be acknowledged in any future case undergoing workup in a similar clinical context.

Finally, a novel molecular finding introduces its own uncertainty. When a previously undescribed variant is identified, the diagnosis cannot rest on genetics alone. Instead, the molecular result must be interpreted alongside phenotype, biochemical pattern, inheritance model, and its predicted functional consequences. Truncating variants affecting the cytoplasmic C-terminal domain, the region encoded by the distal exons of *SLC12A3*, including exon 25, are well established to cause profound loss of NCC function through premature termination or nonsense-mediated mRNA decay, a mechanism fully consistent with the typical biochemical phenotype observed in this patient and with previously described loss-of-function variants in this region^10^.

## Clinicopathological Correlation

The clinicopathological correlation in this case is direct and instructive. The *SLC12A3* gene encodes the NCC cotransporter of the distal convoluted tubule, which is responsible for electroneutral sodium-chloride reabsorption and is also the pharmacological target of thiazide diuretics^1,3^. Loss-of-function variants reduce NCC activity and thereby mimic chronic thiazide exposure. The resulting renal sodium wasting causes mild but persistent extracellular volume contraction, which activates the renin-angiotensin-aldosterone system and secondarily increases aldosterone secretion^2,4,5^. Aldosterone enhances sodium reabsorption in the collecting duct at the expense of potassium and hydrogen ion secretion, explaining the hypokalemia and metabolic alkalosis.

The hypomagnesemia observed in GS is more complex but is thought to reflect impaired magnesium entry through TRPM6 channels in the distal convoluted tubule, likely secondary to altered tubular structure and function produced by defective NCC signaling^3,6^. This mechanism helps explain why magnesium depletion may persist despite supplementation and may be harder to correct than potassium levels, as occurred in this patient.

Hypocalciuria is another characteristic biochemical marker and is useful diagnostically. In GS, increased proximal calcium reabsorption occurs in response to mild volume depletion, while distal sodium transport abnormalities may further favor calcium conservation^3^. This differs from Bartter syndrome, where hypercalciuria or normocalciuria is more typical^9^. Thus, the patient’s very low urinary calcium excretion was not merely an incidental finding.

The molecular result also fits the phenotype. More than 400 to 500 pathogenic variants in *SLC12A3* have been reported, including missense variants, nonsense variants, splice-site changes, small insertions and deletions, and larger rearrangements^4,5,10^. Missense variants are the most frequently identified and may impair NCC trafficking, membrane expression, or transport activity to variable degrees^4,7^. By contrast, truncating mutations such as nonsense or frameshift variants generally produce a more profound loss of function because they lead to premature termination or unstable protein products^4^. The exon 25 out-of-frame deletion identified here is expected to abolish normal NCC function and is therefore fully consistent with the patient’s biochemical phenotype.

The mutational landscape of GS is increasingly recognized as heterogeneous. In addition to classic biallelic *SLC12A3* mutations, Gitelman-like phenotypes have been described in association with variants in other genes and, in selected cases, mitochondrial DNA abnormalities^4^. Compound heterozygosity is common, and genotype-phenotype correlations are imperfect, suggesting that modifier genes, environmental factors, diet, and adaptive renal responses all influence disease expression^1,4,7^. This complexity likely explains why some patients present early with symptomatic disease, whereas others, such as the present patient, remain relatively oligosymptomatic until late adulthood.

## Diagnostic and Therapeutic Implications

This case has several practical implications. GS should remain in the differential diagnosis of persistent hypokalemia in older adults. Age alone should not exclude an inherited tubulopathy. Whenever hypokalemia coexists with metabolic alkalosis, normal or low blood pressure, hypomagnesemia, and hypocalciuria, GS should be actively considered^2,8,9^.

Diagnosis depends on pattern recognition rather than on any isolated abnormality. Hypokalemia is common, but the combination of hypokalemia, hypomagnesemia, metabolic alkalosis, and hypocalciuria is highly informative. A structured approach that excludes gastrointestinal losses, medication effects, and endocrine causes can substantially narrow the possibilities and guide the appropriate confirmatory testing.

Nowadays, genetic testing has become central to modern diagnosis. In atypical presentations, it provides confirmation, allows more confident counseling, and contributes to the expanding catalog of pathogenic *SLC12A3* variants^4,7^. Identification of novel variants is also relevant scientifically, since it improves understanding of the molecular architecture of NCC dysfunction and may eventually refine genotype-phenotype interpretation.

Treatment in GS is mainly supportive and aims to reduce symptoms and minimize complications. Potassium and magnesium supplementation constitutes the cornerstone of therapy, often accompanied by liberal salt intake and use of potassium-sparing agents such as spironolactone or amiloride^1^. In this patient, spironolactone was maintained at 150 mg/day, and oral potassium chloride and magnesium supplementation were added. At three months, serum potassium normalized, whereas hypomagnesemia persisted despite treatment, a common therapeutic challenge in GS, also due to gastrointestinal impairment secondary to medication. Over a 5-year follow-up period, the biochemical profile remained stable, blood pressure was preserved, renal function remained normal, and no neuromuscular or cardiovascular complications were reported.

Long-term management matters because chronic hypokalemia and hypomagnesemia may have important consequences. Persistent potassium depletion has been associated with impaired urinary concentrating ability, tubulointerstitial injury, and hypokalemic nephropathy^11^. Cardiovascular risk is also relevant, particularly the risk of arrhythmia in predisposed patients^12,13^. In addition, chronic hypokalemia may impair insulin secretion and contribute to metabolic disturbances^7^. Recognition and treatment are therefore not merely corrective but potentially preventive.

## Conclusion

This case illustrates the diagnostic reasoning required to identify Gitelman syndrome in an elderly patient with long-standing unexplained hypokalemia. It shows how the integration of chronic clinical history, characteristic biochemical findings, exclusion of acquired causes, and molecular confirmation can transform a nonspecific laboratory abnormality into a precise diagnosis. It also reinforces that inherited tubulopathies should be considered even in late adulthood when the biochemical pattern is suggestive.

The identification of a previously unreported homozygous out-of-frame deletion in exon 25 of *SLC12A3* expands the known mutational spectrum of GS and supports the value of genetic testing in atypical or delayed presentations. Early recognition of GS may facilitate targeted therapy, reduce long-term renal and cardiovascular risks, and improve understanding of genotype-phenotype relationships in this clinically heterogeneous disorder.

## Data Availability

All relevant data are included in the article.
