# Supplementary material for: Late diagnosis of Gitelman syndrome in an elderly patient: identification of a novel SLC12A3 pathogenic variant
Source: J Bras Nefrol. 2026 Sep 14;48(3):e20260105. doi: 10.1590/2175-8239-JBN-2026-0105en (PMC13577598; doi:10.1590/2175-8239-JBN-2026-0105en)
Supplement: Translation

# Translation: Diagnóstico tardio de síndrome de Gitelman em paciente idoso: identificação de uma nova variante patogênica no *SLC12A3*

**DOI:** 10.1590/2175-8239-JBN-2026-0105pt

**Authors:** Lígia Ribeiro, Micael Pompermayer, Catarina Santos, Joana Coutinho

**Affiliations:** 1 Unidade Local de Saúde de Castelo Branco, Serviço de Nefrologia, Castelo Branco, Portugal.

**Keywords:** Síndrome de Gitelman, Tubulopatia Hereditária, Hipocalemia, Testes Genéticos.

## Abstract

A síndrome de Gitelman (SG) é uma tubulopatia hereditária, perdedora de sal e hipocalêmica, causada sobretudo por variantes bialélicas com perda de função no gene *SLC12A3*. Apesar de ser classicamente diagnosticada na adolescência, seu reconhecimento pode ser tardio. Apresentamos o caso de um homem de 74 anos com antecedentes de hipocalemia, cuja avaliação diagnóstica revelou hipocalemia, hipomagnesemia, alcalose metabólica, hipocalciúria e hiperaldosteronismo hiperreninêmico secundário, compatíveis com perda renal de potássio. Dada a apresentação tardia, o principal desafio foi a necessidade de distinguir uma tubulopatia hereditária das causas adquiridas comuns de hipocalemia. Foi realizado teste genético, que identificou uma deleção homozigótica com desvio do quadro de leitura (*out-of-frame*), até então não descrita, no éxon 25 do gene *SLC12A3*, prevendo-se que resulte em perda de função da proteína e, portanto, seja patogênica. O tratamento com suplementação associada à espironolactona permitiu melhoria bioquímica sustentada e estabilidade clínica durante o seguimento a longo prazo. Este caso realça que a SG deve ser considerada em pacientes com hipocalemia crônica inexplicada, independentemente da idade, e que o teste genético é essencial para o diagnóstico definitivo.

## Introdução

A síndrome de Gitelman (SG) é uma tubulopatia perdedora de sal, de trans­missão autossômica recessiva, carac­terizada por hipocalemia, alcalose meta­bólica, hipomagnesemia, hipocalciúria e hiperaldosteronismo hiperreninêmico secundário^1,2^. É considerada a tubulopatia hereditária mais frequente, com uma prevalência estimada entre 1 em 10.000 e 1 em 40.000 indivíduos, embora a prevalência real possa estar subestimada, uma vez que os pacientes com formas leves permanecem frequentemente sem diagnóstico^1^. A doença resulta, na maioria dos casos, de variantes patogênicas bialélicas no gene *SLC12A3*, que codifica o cotransportador de sódio-cloro sensível às tiazidas (NCC), localizado na membrana apical das células do túbulo contornado distal^1,3^.

A perda da atividade do NCC compromete a reabsorção de sódio e cloro, conduzindo a uma leve contração do volume extracelular. Esta perda crônica de sal ativa o sistema renina-angiotensina-aldosterona (SRAA), promovendo a reabsorção distal de sódio em troca da secreção de potássio e de íons de hidrogênio, resultando assim em hipocalemia e alcalose metabólica^2,4,5^. Pensa-se que a hipomagnesemia associada seja secundária a uma alteração do transporte de magnésio, particularmente por meio da diminuição da expressão ou da disfunção da reabsorção de magnésio mediada pelo TRPM6 no túbulo contornado distal^3,6^. A hipocalciúria, outro achado característico da SG, provavelmente reflete o aumento da reabsorção tubular proximal de cálcio em resposta à leve depleção de volume e às alterações do transporte tubular distal^3^.

O fenótipo da SG é heterogêneo. Alguns pacientes são essencialmente assintomáticos, sendo diagnosticados apenas após a detecção incidental de alterações laboratoriais, ao passo que outros desenvolvem fadiga, fraqueza muscular, cãibras, avidez por sal, sede, nictúria, parestesias, tetania, rabdomiólise, condrocalcinose ou arritmias cardíacas^7,8^. A gravidade das manifestações depende não só do grau de distúrbio eletrolítico, mas também da idade, da ingestão alimentar, das comorbidades, de doença intercorrente e do contexto genético específico do paciente^1,7^. Embora a SG seja frequentemente diagnosticada na adolescência ou no início da idade adulta, pode ocorrer um diagnóstico tardio, sobretudo em indivíduos com sintomas leves ou alterações bioquímicas flutuantes.

Na prática clínica, a hipocalemia persistente é muito mais frequentemente atribuída a causas adquiridas do que a tubulopatias hereditárias. As perdas gastrointestinais, o uso de diuréticos ou laxantes, as doenças endócrinas e as causas renais secundárias são geralmente as primeiras a ser consideradas^8^. Como consequência, a SG pode não ser suspeitada até que se torne evidente uma constelação bioquímica característica. Isso é particularmente verdadeiro em idosos, nos quais os clínicos podem estar menos predispostos a considerar uma doença hereditária. O diagnóstico diferencial inclui também a síndrome de Bartter e outras doenças tubulares renais, exigindo uma interpretação cuidadosa^9^.

Este relato utiliza o caso de um paciente idoso com hipocalemia inexplicada de longa data para ilustrar uma discussão clínico-patológica estruturada, centrada no raciocínio diagnóstico, nos diagnósticos diferenciais relevantes, na correlação clínico-patológica e nas implicações terapêuticas. Amplia igualmente o espectro mutacional da SG ao descrever uma variante do *SLC12A3* até então não descrita.

## Raciocínio Diagnóstico

Um homem de 74 anos, obeso (IMC de 31,2 kg/m^2^), com antecedentes de dislipidemia e gastrite atrófica, foi encaminhado a uma consulta de medicina interna após a detecção de hipocalemia moderada (potássio sérico de 2,6 mmol/L) durante uma ida ao serviço de urgência por vômitos agudos, atribuídos a presumível gastroenterite viral. Sua medicação habitual incluía espironolactona, tansulosina e rosuvastatina. A revisão dos registros prévios demonstrou que uma hipocalemia leve havia sido documentada repetidamente na atenção primária à saúde ao longo dos 10 anos anteriores. À data da avaliação ambulatorial, os vômitos já haviam se resolvido completamente. Embora as perdas gastrointestinais agudas pudessem justificar uma depleção transitória de potássio, não explicavam um padrão de hipocalemia persistente com uma década de evolução. O paciente descrevia sede e nictúria de longa data, mas negava fraqueza muscular, cãibras, parestesias, palpitações, síncope ou episódios sugestivos de tetania. Não havia história sugestiva de transtornos do comportamento alimentar, diarreia, abuso de laxantes ou uso dissimulado de diuréticos. A exclusão do uso dissimulado de laxantes e diuréticos baseou-se na história clínica e na ausência de qualquer prescrição de diuréticos de alça ou tiazídicos nos registros de medicação do paciente ao longo da década anterior, em conjunto com a excreção urinária de cloro persistentemente baixa (92 mmol/24 h, abaixo do limite inferior da normalidade de 110 mmol/24 h), o que seria atípico em caso de uso ativo de diuréticos. Os antecedentes familiares eram irrelevantes.

O primeiro passo diagnóstico foi distinguir entre perda de potássio extrarrenal e renal. Em um paciente com hipocalemia crônica, a alcalose metabólica levanta de imediato várias possibilidades, incluindo vômitos, uso de diuréticos, excesso de mineralocorticoides e tubulopatias hereditárias perdedoras de sal^8^. A ausência de sintomas gastrointestinais em curso, a cronicidade da alteração e a ausência de perda de potássio claramente relacionada a medicamentos tornaram mais provável a perda renal de potássio.

A avaliação laboratorial sob a medicação habitual confirmou hipocalemia e hipomagnesemia persistentes, associadas à alcalose metabólica. Os resultados obtidos são apresentados na Tabela 1. A função renal e a função tireoidiana eram normais. Os níveis plasmáticos de renina e de aldosterona estavam elevados, indicando um hiperaldosteronismo hiperreninêmico secundário, e não um excesso primário de aldosterona. Este perfil sugeria fortemente uma perda crônica de sódio com depleção efetiva de volume.

**Tabela 1:** Achados laboratoriais na admissão de um paciente com SG

Análise laboratorial (unidades)	Valores obtidos	Valores de referência
Gasometria arterial		
pH	7,421	7,350–7,450
pO_2_ (mmHg)	88	75–100
pCO_2_ (mmHg)	36,8	35–45
Potássio (mmol/L)	3,31	3,5–4,5
Cálcio (mmol/L)	1,27	1,15–1,30
Bicarbonato (mmol/L)	23	18–22
Bioquímica e hemograma		
Hemoglobina (Hb) (g/dL)	16,7	13,0–17,0
Leucócitos (10^3^/µL)	8,29	4,0–10,0
Plaquetas (10^3^/µL)	298	150–400
Ureia sérica (mg/dL)	33,3	19,3–49,3
Creatinina sérica (mg/dL)	0,77	0,70–1,30
Sódio (mmol/L)	137	136–145
Potássio (mmol/L)	3,4	3,6–5,1
Magnésio (mmol/L)	1,49	1,60–2,60
Hormônio estimulante da tireoide (µUI/mL)	1,68	0,40–4,40
Cortisol (ng/dL)	22	4,30–22,40
Hormônio adrenocorticotrófica (pg/mL)	24	9–52
Renina ativa (µU/mL)	278	7–76
Aldosterona (pg/mL)	1355	40–310
Urina de 24 horas		
Volume (mL)	2400	–
Potássio (mmol/24 h)	127,2	25,0–125,0
Sódio (mmol/24 h)	88,3	40,0–220,0
Cloro (mmol/24 h)	92	110,0–250,0
Cálcio (g/24 h)	0,06	0,10–0,30

A coleta de urina de 24 horas revelou hipocalciúria, com uma excreção fracionada de cálcio de 0,0045%. Este achado foi particularmente informativo. No contexto de alcalose metabólica hipocalêmica, a hipocalciúria está classicamente associada à SG e ajuda a distingui-la da síndrome de Bartter, na qual a excreção urinária de cálcio é habitualmente normal ou aumentada^9^. A presença de hipomagnesemia apoiou ainda mais o diagnóstico de SG, uma vez que a depleção de magnésio é frequente e muitas vezes proeminente nesta doença^2,3^.

Nesta fase, o diagnóstico mais provável era a síndrome de Gitelman. Contudo, ainda era necessário excluir explicações alternativas. O hiperaldosteronismo primário era improvável, uma vez que a renina estava elevada e não suprimida. O hipercortisolismo não foi apoiado pelos níveis de cortisol. A estenose da artéria renal, que também pode causar hiperaldosteronismo hiperreninêmico, foi excluída por tomografia computadorizada abdominal com angiografia. Não foi identificada qualquer massa adrenal ou outra massa intra-abdominal. A imagem também não mostrou nefrocalcinose, um achado que, se presente, poderia apontar para outras doenças tubulares ou para um desequilíbrio mineral de longa data.

A eletrocardiografia mostrou ritmo sinusal e a ecocardiografia transtorácica demonstrou apenas insuficiência aórtica leve. Esses achados eram tranquilizadores diante do risco de arritmia associado à hipocalemia crônica, mas não diminuíam a necessidade de esclarecer a causa subjacente.

Em conjunto, a hipocalemia persistente, a alcalose metabólica, a hipomagnesemia, a hipocalciúria, a pressão arterial normal e o hiperaldosteronismo hiperreninêmico secundário constituíam um padrão bioquímico altamente coerente com a SG. Dado que a apresentação foi incomumente tardia e não havia história familiar disponível, procedeu-se à confirmação genética, tanto para assegurar o diagnóstico como para caracterizar melhor o defeito subjacente.

Após obtenção do consentimento informado, o sequenciamento de nova geração numa plataforma Illumina identificou uma deleção homozigótica com desvio do quadro de leitura (*out-of-frame*) do éxon 25 do gene *SLC12A3*, até então não descrita: c.(2883 + 1_2884-1)_(2951 + 1_2952-1)del. A variante estava ausente de bases de dados populacionais, incluindo a gnomAD e a *Database of Genomic Variants*. Dado o seu previsível efeito disruptivo sobre o quadro de leitura e a consequente perda da função normal da proteína, foi considerada patogênica e confirmou o diagnóstico de síndrome de Gitelman. Não foi realizada triagem familiar, pois o paciente não possuía familiares de primeiro grau disponíveis para avaliação.

Este perfil bioquímico manteve-se estável ao longo de um período de seguimento de 5 anos, conforme apresentado na Tabela 2, sem complicações neuromusculares ou cardiovasculares registradas e com valores de pressão arterial estáveis.

**Tabela 2:** Valores séricos anuais de potássio, magnésio e creatinina ao longo de 5 anos de seguimento

	Potássio sérico (mmol/L)	Magnésio sérico (mg/dL)	Creatinina sérica (mg/dL)
Ano 1	3,60	1,22	0,79
Ano 2	3,68	1,30	0,81
Ano 3	3,63	1,31	0,80
Ano 4	3,71	1,24	0,79
Ano 5	3,68	1,28	0,80

## Desafios Clínicos

Este caso ilustra diversas razões pelas quais a SG pode permanecer sem ser reconhecida durante muitos anos. Embora a SG seja uma doença monogênica, sua expressão clínica varia desde alterações bioquímicas quase silenciosas até doença sintomática com comprometimento substancial, traduzindo uma elevada variabilidade fenotípica^1,7^. Neste paciente, a ausência de queixas neuromusculares ou cardiovasculares manifestas contribuiu provavelmente para um baixo grau de suspeita clínica durante muitos anos.

Como a SG está tradicionalmente associada a pacientes mais jovens, os clínicos podem não a considerar prontamente em um idoso que se apresenta com hipocalemia crônica. No entanto, o defeito genético está presente ao longo de toda a vida, e a idade ao diagnóstico depende mais da carga sintomática e do grau de suspeição diagnóstica do que do momento de início da doença.

Outros desafios envolveram a interpretação dos dados urinários e bioquímicos no contexto do tratamento. O paciente já se encontrava sob espironolactona à época da avaliação, e a suplementação subsequente também influenciou as medições de seguimento. Na prática do mundo real, as investigações são frequentemente realizadas em condições não ideais, e o raciocínio diagnóstico tem de integrar dados imperfeitos, em vez de se basear em um único resultado analítico sem fatores de confusão. A espironolactona pode ter corrigido parcialmente os níveis de potássio, atenuado a resposta da aldosterona e estimulado ainda mais a secreção de renina por meio de um hiperreninismo reativo. Apesar disso, o padrão bioquímico global com hipocalemia persistente, hipomagnesemia, hipocalciúria e renina elevada com hiperaldosteronismo secundário manteve-se altamente compatível com a síndrome de Gitelman. É importante salientar que o diagnóstico foi, em última análise, assegurado pela confirmação molecular, que é independente dos fatores de viés farmacológicos. Esta limitação é particularmente relevante e deve ser reconhecida em qualquer caso futuro submetido à investigação em um contexto clínico semelhante.

Por fim, um achado molecular inédito acarreta sua própria incerteza. Quando é identificada uma variante não descrita previamente, o diagnóstico não pode se basear apenas na genética. Pelo contrário, o resultado molecular deve ser interpretado em conjunto com o fenótipo, o padrão bioquímico, o modelo de hereditariedade e as consequências funcionais previstas. As variantes truncantes que afetam o domínio C-terminal citoplasmático — a região codificada pelos éxons distais do *SLC12A3*, incluindo o éxon 25 — estão bem estabelecidas como causa de perda profunda da função do NCC, por terminação prematura ou por degradação do mRNA mediada por mutações sem sentido (NMD), um mecanismo totalmente compatível com o fenótipo bioquímico típico observado neste paciente e com as variantes com perda de função previamente descritas nesta região^10^.

## Correlação Clínico-Patológica

A correlação clínico-patológica neste caso é direta e instrutiva. O gene *SLC12A3* codifica o cotransportador NCC do túbulo contorcido distal, responsável pela reabsorção eletroneutra de sódio-cloro, que constitui também o alvo farmacológico dos diuréticos tiazídicos^1,3^. As variantes com perda de função reduzem a atividade do NCC, mimetizando assim uma exposição crônica às tiazidas. A consequente perda renal de sódio provoca uma contração leve, mas persistente, do volume extracelular, que ativa o sistema renina-angiotensina-aldosterona e, secundariamente, aumenta a secreção de aldosterona^2,4,5^. A aldosterona intensifica a reabsorção de sódio no túbulo coletor à custa da secreção de potássio e de íons de hidrogênio, explicando a hipocalemia e a alcalose metabólica.

A hipomagnesemia observada na SG é mais complexa, mas pensa-se que reflita um comprometimento da entrada de magnésio através dos canais TRPM6 no túbulo contorcido distal, provavelmente secundário às alterações da estrutura e da função tubulares produzidas pela sinalização deficiente do NCC^3,6^. Este mecanismo ajuda a explicar por que razão a depleção de magnésio pode persistir apesar da suplementação e pode ser mais difícil de corrigir do que os níveis de potássio, como observado neste paciente.

A hipocalciúria é outro marcador bioquímico característico e é útil do ponto de vista diagnóstico. Na SG, ocorre um aumento da reabsorção proximal de cálcio em resposta à leve depleção de volume, enquanto as alterações do transporte distal de sódio podem favorecer ainda mais a conservação de cálcio^3^. Isso difere da síndrome de Bartter, na qual a hipercalciúria ou a normocalciúria são mais típicas^9^. Assim, a excreção urinária de cálcio muito baixa do paciente não constituía um achado meramente incidental.

O resultado molecular também é concordante com o fenótipo. Foram descritas mais de 400 a 500 variantes patogênicas no *SLC12A3*, incluindo variantes *missense*, variantes *nonsense*, alterações do sítio de *splicing*, pequenas inserções e deleções, e rearranjos de maiores dimensões^4,5,10^. As variantes *missense* são as mais frequentemente identificadas e podem comprometer, em graus variáveis, o tráfego intracelular do NCC, sua expressão na membrana ou a atividade de transporte^4,7^. Em contrapartida, as mutações truncantes, como as variantes *nonsense* ou *frameshift*, produzem geralmente uma perda de função mais profunda, por conduzirem à terminação prematura ou a produtos proteicos instáveis^4^. Prevê-se que a deleção com desvio do quadro de leitura do éxon 25 aqui identificada elimine a função normal do NCC, sendo, por conseguinte, totalmente compatível com o fenótipo bioquímico do paciente.

O panorama mutacional da SG é cada vez mais reconhecido como heterogêneo. Além das clássicas mutações bialélicas do *SLC12A3*, foram descritos fenótipos semelhantes à SG (*Gitelman-like*) em associação com variantes em outros genes e, em casos selecionados, com anomalias do DNA mitocondrial^4^. A heterozigose composta é frequente, e as correlações genótipo-fenótipo são imperfeitas, sugerindo que genes modificadores, fatores ambientais, dieta e respostas renais adaptativas influenciam a expressão da doença^1,4,7^. Essa complexidade explica provavelmente por que razão alguns pacientes se apresentam precocemente com doença sintomática, ao passo que outros, como o presente paciente, permanecem relativamente oligossintomáticos até a idade adulta avançada.

## Implicações Diagnósticas e Terapêuticas

Este caso apresenta diversas implicações práticas. A SG deve permanecer no diagnóstico diferencial da hipocalemia persistente em idosos. A idade, por si só, não deve excluir uma tubulopatia hereditária. Sempre que a hipocalemia coexista com alcalose metabólica, pressão arterial normal ou baixa, hipomagnesemia e hipocalciúria, a SG deve ser ativamente considerada^2,8,9^.

O diagnóstico depende do reconhecimento de um padrão, e não de qualquer alteração isolada. A hipocalemia é frequente, mas a combinação de hipocalemia, hipomagnesemia, alcalose metabólica e hipocalciúria é altamente informativa. Uma abordagem estruturada, que exclua perdas gastrointestinais, efeitos da medicação e causas endócrinas, pode reduzir substancialmente as possibilidades e orientar o teste confirmatório adequado.

Atualmente, o teste genético tornou-se central no diagnóstico moderno. Nas apresentações atípicas, fornece confirmação, permite um aconselhamento mais seguro e contribui para o catálogo em expansão de variantes patogênicas do *SLC12A3* ^4,7^. A identificação de novas variantes é também relevante do ponto de vista científico, uma vez que melhora a compreensão da arquitetura molecular da disfunção do NCC e pode, no futuro, refinar a interpretação genótipo-fenótipo.

O tratamento da SG é sobretudo de suporte e visa reduzir os sintomas e minimizar as complicações. A suplementação de potássio e de magnésio constitui a pedra angular da terapêutica, sendo frequentemente acompanhada por uma ingestão liberal de sal e pelo uso de agentes poupadores de potássio, como a espironolactona ou a amilorida^1^. Neste paciente, a espironolactona foi mantida em 150 mg/dia e foram associados suplementos orais de cloreto de potássio e de magnésio. Aos três meses, o potássio sérico normalizou, ao passo que a hipomagnesemia persistiu apesar do tratamento, um desafio terapêutico comum na SG, também devido ao comprometimento gastrointestinal secundário à medicação. Ao longo de um período de seguimento de 5 anos, o perfil bioquímico manteve-se estável, a pressão arterial foi preservada, a função renal permaneceu normal e não foram observadas complicações neuromusculares ou cardiovasculares.

A abordagem a longo prazo é importante, uma vez que a hipocalemia e a hipomagnesemia crônicas podem ter consequências relevantes. A depleção persistente de potássio tem sido associada a comprometimento da capacidade de concentração urinária, à lesão tubulointersticial e à nefropatia hipocalêmica^11^. O risco cardiovascular é igualmente relevante, sobretudo o risco de arritmia em pacientes predispostos^12,13^. Além disso, a hipocalemia crônica pode comprometer a secreção de insulina e contri­buir para distúrbios metabólicos^7^. O reconhecimento e o tratamento não são, por conseguinte, meramente corretivos, mas potencialmente preventivos.

## Conclusão

Este caso ilustra o raciocínio diagnóstico necessário para identificar a síndrome de Gitelman em um paciente idoso com hipocalemia inexplicada de longa data. Demonstra como a integração de uma história clínica crônica, de achados bioquímicos característicos, da exclusão de causas adquiridas e da confirmação molecular pode transformar uma alteração laboratorial inespecífica em um diagnóstico preciso. Reforça igualmente que as tubulopatias hereditárias devem ser consideradas mesmo em idade adulta avançada quando o padrão bioquímico é sugestivo.

A identificação de uma deleção homozigótica com desvio do quadro de leitura, até então não descrita, no éxon 25 do *SLC12A3* amplia o espectro mutacional conhecido da SG e apoia o valor do teste genético nas apresentações atípicas ou tardias. O reconhecimento precoce da SG pode facilitar uma terapêutica dirigida, reduzir os riscos renais e cardiovasculares a longo prazo e melhorar a compreensão das relações genótipo-fenótipo nessa doença clinicamente heterogênea.

## Data Availability

Todos os dados relevantes estão incluídos no artigo.
